# Response: Commentary: Case Report: Hyperbilirubinemia in Gilbert Syndrome Attenuates Covid-19-Induced Metabolic Disturbances

**DOI:** 10.3389/fcvm.2021.738798

**Published:** 2021-10-05

**Authors:** Hayder M. Al-kuraishy, Ali I. Al-Gareeb, Saleh M. Abdullah, Natália Cruz-Martins, Gaber El-Saber Batiha

**Affiliations:** ^1^Department of Clinical Pharmacology and Medicine, College of Medicine, Al-Mustansiriya University, Baghdad, Iraq; ^2^Department of Medical Laboratory Technology, Faculty of Applied Medical Sciences, Jazan University, Jazan, Saudi Arabia; ^3^Faculty of Medicine, University of Porto, Porto, Portugal; ^4^Institute for Research and Innovation in Health (i3S), University of Porto, Porto, Portugal; ^5^Institute of Research and Advanced Training in Health Sciences and Technologies (CESPU), Gandra, Portugal; ^6^Department of Pharmacology and Therapeutics, Faculty of Veterinary Medicine, Damanhour University, Damanhour, Egypt

**Keywords:** Gilbert syndrome (GS), SARS-CoV-2 (2019-nCoV), hyperbilirubinemia, COVID - 19, Crigler–Najjar syndrome type II

This is in response to the letter by Minucci et al. (1) addressing our recent article published in *Frontiers in Cardiovascular Medicine* (2). In the commentary, the authors suspected that the reported case was Crigler–Najjar syndrome type II (CNS-II) and not Gilbert syndrome (GS), based on the level of total serum bilirubin (TSB) and unconjugated fraction. CNS-II is a rare autosomal recessive disorder due to a mutation in the UGT1A1 gene, whose mutation can even cause other metabolic disorders, like CNS-I and GS, resulting in a reduction of the UDP-glucuronosyl transferase function, which is responsible for the conjugation of bilirubin (3). In addition, CNS-II is usually identified with persistent jaundice in the neonate and early childhood and very rarely in adults.

The TSB level in CNS-II patients commonly ranges from 10 to 20 mg/dL (mostly unconjugated), and increased up to 40 mg/dL during exacerbation and partly responds to the effect of phenobarbitone within 2–3 weeks (4). In a study, Kumar and colleagues (5) illustrated that CNS-II is an unwanted cause of jaundice in adults. In contrast, the prevalence of GS is between 4 and 16% for the general population compared to 1 per million for CNS-II. Moreover, hyperbilirubinemia in GS is completely normalized following phenobarbitone therapy and rarely exceeds 6 mg/dL (mostly unconjugated) (6). However, the serum TSB level in our reported case had a slightly higher serum TSB level (6.5 mg/dL), which might be due to the inflammatory burden caused by COVID-19. Skierka et al. (7) and Sood et al. (8) have shown that GS cases can have higher bilirubin levels than usually reported, despite that the TSB level varies continuously from GS to CNS-II, depending on genotypes. In fact, because of the combination of polymorphisms and mutations, many patients experience intermediate TSB level between the two syndromes (9).

These findings rule out of CNS-II as a cause of inherited hyperbilirubinemia in the present study. Indeed, the case report presented is well-diagnosed since the age of 4 years by genetic analysis; however, this genetic analysis was not performed for other family members, as we mentioned in the limitations to the study. TSB alone is considered a hurdle in differentiating GS from CNS-II; nonetheless, the reduction in TSB level following phenobarbitone is regarded as a diagnostic clincher in differentiating GS (complete response) from CNS-II (partial response).

It is also worth noting that COVID-19 therapies used in our reported case, such as montelukast and prophylactic antibiotics (ceftriaxone), may increase the serum bilirubin level (5, 10). Besides, a pooled analysis study confirmed that the TSB level is associated and correlated with COVID-19 severity due to the alteration in bilirubin dynamics by an unknown mechanism (11).

Taken together, these points endorse the original diagnosis of GS, but not CNS-II, in contrast to the suggestion of Minucci et al. in their commentary.

## Author Contributions

All authors have significantly contributed to this work.

## Conflict of Interest

The authors declare that the research was conducted in the absence of any commercial or financial relationships that could be construed as a potential conflict of interest.

## Publisher's Note

All claims expressed in this article are solely those of the authors and do not necessarily represent those of their affiliated organizations, or those of the publisher, the editors and the reviewers. Any product that may be evaluated in this article, or claim that may be made by its manufacturer, is not guaranteed or endorsed by the publisher.
